# Free-Breathing 3D Imaging of Right Ventricular Structure and Function Using Respiratory and Cardiac Self-Gated Cine MRI

**DOI:** 10.1155/2015/819102

**Published:** 2015-06-21

**Authors:** Yanchun Zhu, Jing Liu, Jonathan Weinsaft, Pascal Spincemaille, Thanh D. Nguyen, Martin R. Prince, Shanglian Bao, Yaoqin Xie, Yi Wang

**Affiliations:** ^1^Institute of Biomedical and Health Engineering, Shenzhen Institutes of Advanced Technology, Chinese Academy of Sciences, 1068 Xueyuan Avenue, Shenzhen University Town, Nanshan District, Shenzhen 518055, China; ^2^Beijing City Key Lab of Medical Physics and Engineering, School of Physics, Peking University, 201 Chengfu Road, Haidian District, Beijing 100871, China; ^3^Department of Radiology, Weill Cornell Medical College, 515 East 71th Street, New York, NY 10021, USA; ^4^Division of Cardiology, Department of Medicine, Weill Cornell Medical College, 520 East 70th Street, New York, NY 10065, USA

## Abstract

Providing a movie of the beating heart in a single prescribed plane, cine MRI has been widely used in clinical cardiac diagnosis, especially in the left ventricle (LV). Right ventricular (RV) morphology and function are also important for the diagnosis of cardiopulmonary diseases and serve as predictors for the long term outcome. The purpose of this study is to develop a self-gated free-breathing 3D imaging method for RV quantification and to evaluate its performance by comparing it with breath-hold 2D cine imaging in 7 healthy volunteers. Compared with 2D, the 3D RV functional measurements show a reduction of RV end-diastole volume (RVEDV) by 10%, increase of RV end-systole volume (RVESV) by 1.8%, reduction of RV systole volume (RVSV) by 21%, and reduction of RV ejection fraction (RVEF) by 12%. High correlations between the two techniques were found (RVEDV: 0.94; RVESV: 0.85; RVSV: 0.95; and RVEF: 0.89). Compared with 2D, the 3D image quality measurements show a small reduction in blood SNR, myocardium-blood CNR, myocardium contrast, and image sharpness. In conclusion, the proposed self-gated free-breathing 3D cardiac cine imaging technique provides comparable image quality and correlated functional measurements to those acquired with the multiple breath-hold 2D technique in RV.

## 1. Introduction

Cardiac magnetic resonance (CMR) is a widely used noninvasive imaging method for depicting cardiac structure, function, perfusion, and viability [1]. Cine MRI can capture the cyclic contraction and relaxation of the heart, enabling the evaluation of ventricular and valvular function as well as shunt detection [2]. Cardiac cine images are conventionally acquired using a breath-hold 2D balanced steady-state free procession (SSFP) pulse sequence, which can provide accurate and reproducible volume quantification [3] of both the left ventricle (LV) [4, 5] and the right ventricle (RV) [6–8]. However, the accuracy can be compromised by the slice misregistration due to inconsistent breath-holding levels during subsequent 2D scans and also by the slice gap often used to shorten the number of required breath-holds in less cooperative patients. Breath-hold 3D SSFP cine MRI has been developed to overcome these challenges by providing contiguous spatial coverage without gap and eliminating slice misregistration. However, this approach requires a long breath-hold, which is not suitable for older patients, particularly those with cardiopulmonary diseases. A major limitation of breath-hold cine MRI in general is spatial resolution, which is constrained by the length of the breath-hold. Finally, compared to free breathing, breath holding alters the intrathoracic pressure which can impact right ventricular filling.

Recently, respiratory and cardiac self-gated cardiac cine MRI pulse sequences have emerged as promising imaging approaches for achieving higher resolution during free breathing [9, 10]. While the utility of respiratory and cardiac self-gated cardiac cine MRI for LV imaging has been demonstrated, its application to the assessment of RV appears quite limited due to its thin wall structure. RV morphology and function have been increasingly recognized as important cardiac parameters in the diagnosis and treatment of patients with cardiopulmonary diseases [7] and especially congenital heart disease [11]. The purpose of this study was to develop a self-gated free-breathing 3D SSFP cine imaging method for RV quantification and to evaluate its performance by comparing with breath-hold 2D SSFP cine imaging in healthy volunteers.

## 2. Materials and Methods

### 2.1. Self-Gated Pulse Sequence Design

**Figure 1:**
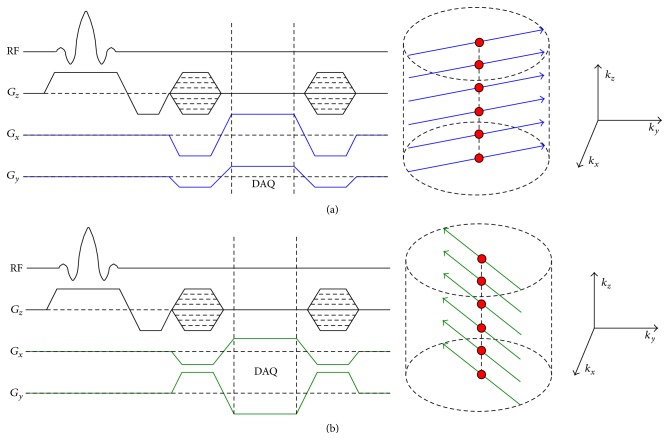
SSFP pulse sequence collects a profile with multiple slice encoding lines for a specific projection angle (blue lines in (a)) and changed projection angle after finishing the last profile (green lines in (b)).

Figure 1 shows the pulse sequence diagram of the implemented self-gated 3D SSFP cine pulse sequence with hybrid radial *k*-space sampling (Cartesian sampling along slice encoding direction (*k*_*z*_) and radial sampling in the *k*_*x*_-*k*_*y*_ plane) [12, 13]. All slice encodes for a given projection angle are acquired sequentially (Figure 1(a)) and named as a profile. The acquisition then switches to the next projection angle (Figure 1(b)) and acquires the next profile. Each profile including *n*_*z*_ slice encodes spends the time of *n*_*z*_ TR. The projection angle is increased by the golden angle of 111.2° to generate approximately uniform *k*-space distribution of the projections at any time point, allowing for robust sliding window reconstructions to achieve the desired temporal resolution and/or undersampling artifacts [14].

### 2.2. Image Reconstruction

**Figure 2:**
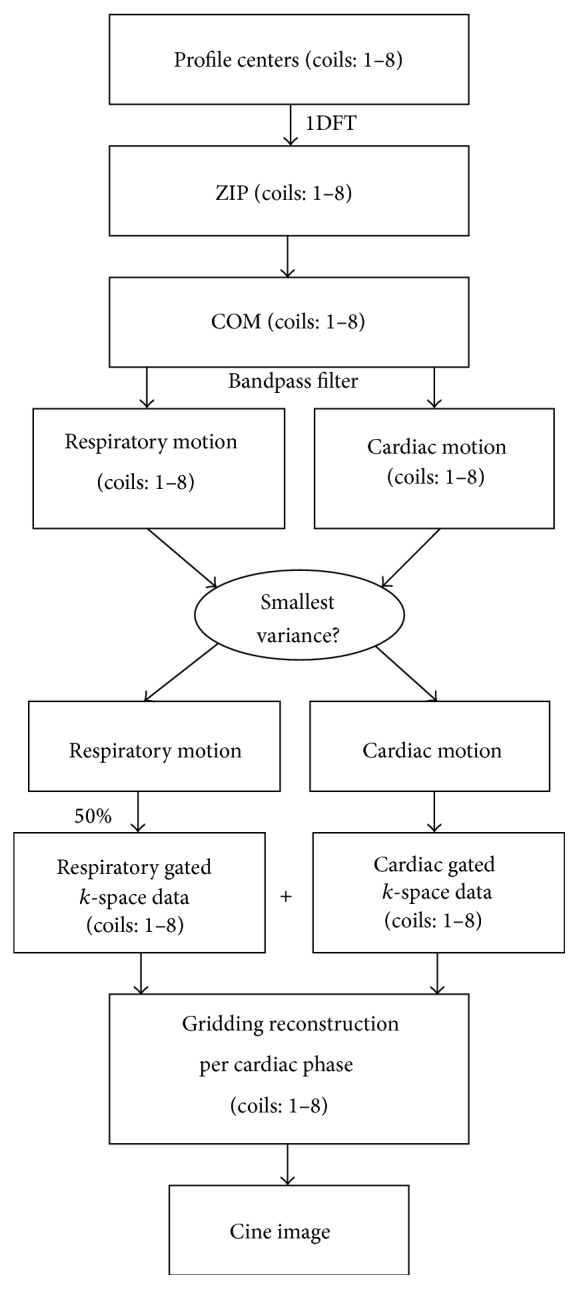
Block diagram of the algorithm used in self-gated reconstruction. Profile centers are used to derive self-gating signals. Respiratory and cardiac self-gating signals are used to classify profiles before using gridding reconstruction for each cardiac phase.

The flow chart of self-gating image reconstruction is shown in Figure 2. The profile centers, shown as red points in Figure 1, were used to extract the self-gating signal of both respiratory and cardiac motions. As described in [12], 1D Fourier transform of profile centers can be used to derive imaging volume projection along the *z*-axis, which is called *Z*-intensity projection (ZIP). The center of mass (COM) of each ZIP contains mixed respiratory and cardiac motions that occur during the data acquisition. Since these motions are known to have different frequency contents (0.1–0.5 Hz for respiratory motion and 0.6–3 Hz for cardiac motion) [15], they can be separated by using band pass filter. The coil element with the smallest variance of the detected R-R intervals was selected for cardiac gating and, similarly, the coil element with the smallest variance of respiratory peak or valley positions was chosen for respiratory gating. This technique was capable of tracking both respiratory and cardiac motions [12].

**Figure 3:**
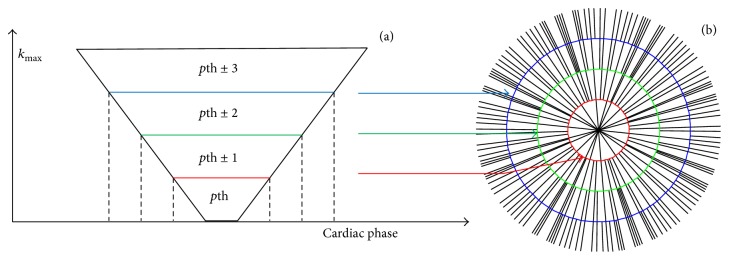
Schematic diagram of tornado temporal filter in sliding window reconstruction of *p*th cardiac phase (a) and corresponding *k*-space distribution (b).

The detected respiratory and cardiac motion signals were used to determine the respiratory position and cardiac phase associated with each profile. A respiratory histogram was calculated and only data acquired within a given window (50% of acquired data) around the peak of the histogram were used for image reconstruction. The profiles detected with the same cardiac phase number, according to the self-gated cardiac motion signal, were used for reconstruction. Sliding window reconstruction with tornado temporal filter [16] (Figure 3(a)) was applied to decrease the streaking artifacts caused by undersampling in the *k*_*x*_-*k*_*y*_ plane. The temporal aperture was the specified cardiac phase (*p*th) beginning at the lowest spatial frequency and increasing linearly to the highest spatial frequency (Figure 3(a)). The corresponding *k*-space distribution for the *p*th cardiac phase reconstruction was depicted in Figure 3(b). The proportion of high spatial frequency *k*-space data shared from neighboring cardiac phases (one-third of the number of cardiac phases) was related to cardiac phase distance. Temporal resolution of each cardiac phase image was calculated as center period of tornado window, which spent *n*_*z*_TR. Images were reconstructed from radial *k*-space data using gridding algorithm [17] with Kaiser-Bessel window as a gridding kernel.

### 2.3. Human Imaging Experiment

Cardiac cine MRI was performed in seven healthy volunteers (32 ± 7 yo, 5 male and 2 female) using a 1.5 T GE HDx scanner (maximum gradient amplitude 33 mT/m, slew-rate 120T/m/s, Excite 14M5 software; GE Healthcare, Waukesha, WI, USA). The study was approved by the local institutional review board at Weill Cornell Medical College, and written informed consent was obtained from all subjects. An eight-channel cardiac phased-array coil was used for signal reception. Both 2D and 3D cine MRI were performed in each subject in randomized order. The typical cine imaging parameters were as follows: (1) breath-hold multislice 2D cine: TR/TE = 3.5/1.2 ms, flip angle = 60°, BW = ±125 kHz, FOV = 31 cm, imaging matrix = 256 × 192 (reconstructed to 256 × 256), slice thickness/gap = 7/3 mm, number of slices = 12–14, measured spatial resolution = 1.2 × 1.6 × 7 mm^3^, reconstructed spatial resolution = 1.2 × 1.2 × 7 mm^3^, views per segment = 24, temporal resolution = 84 ms, and number of reconstructed phases = 28 by view sharing for visualization purposes, scan time about 5 min (including nearly 2.5 min total rest time between consecutive breath-holds); (2) free-breathing self-gated 3D cine: TR/TE = 4.4/1.3 ms, flip angle = 40°, BW = ±125 kHz, FOV = 31 cm, reconstructed image matrix = 256 × 256, slice thickness = 7 mm (no gap), number of slices = 14, measured and reconstructed spatial resolutions = 1.2 × 1.2 × 7 mm^3^, and temporal resolution = 61.6 ms, scan time about 5 min (to match 2D cine acquisition time). RV images were acquired in the short-axis view parallel to the mitral valve from the tricuspid valve to the pulmonic valve annulus.

### 2.4. Data Analysis

RV contours were traced by an experienced physician and RV volumes, from which RV ejection fraction (RVEF) was calculated, were measured by manual planimetry at end-diastole (RVEDV) and end-systole (RVESV) using a modified Simpson's rule. Technical challenges associated with manual RV contouring are related to the thinness of the RV wall, wall trabeculations, infundibulum and pulmonary valve level, separation between RV and right atrium in basal slices, and protrusion of basal structures, such as the initial ascending aorta, all of which can lead to partial volume effect. If the pulmonary valve was evident in the basal slice, both in end-diastole and end-systole, only the portion of the volume below the level of the pulmonary valve was included. For the inflow part of the RV, the blood volume was excluded from the RV volume if the surrounding wall was thin and not trabeculated, as it was considered to be in the right atrium. To assess the agreement between 2D and 3D cine imaging, linear regression and Bland-Altman analysis were used [18]. A two-sided Wilcoxon signed-rank test was used to assess the difference between the two methods. The Pearson correlation coefficient was calculated to assess the correlation between the two methods. *P* value < 0.05 was considered statistically significant.

Image quality measures, including blood SNR, myocardium-blood contrast, CNR, and image sharpness, were calculated from a mid-ventricular slice for each volunteer. Blood SNR was calculated as the ratio of the average blood signal measured in the RV cavity to the standard deviation of background signals. Myocardium-blood contrast was calculated according to

(1) $$\begin{array}{c} Contrast=\frac{S_{blood}-S_{myocardium}}{\left|S_{blood}\right|+\left|S_{myocardium}\right|}\times 100\%, \end{array}$$

where *S*_blood_ and *S*_myocardium_ are the average signal intensities of the blood and myocardium, respectively. This relative measure gives a contrast range of 0-1 [19]. Compared with the contrast-to-noise ratio (CNR), the contrast parameter can directly reflect the contrast of two tissues by omitting the background noise difference. Image sharpness was used to evaluate the interface of the RV myocardium and the RV blood pool. Four signal profiles, evenly spaced around the RV circumference and positioned across the endocardial border of RV, were measured from end-diastole and end-systole mid-RV images. The local maximum (*I*_max_) and minimum (*I*_min_) intensity values across the endocardial border were determined, from which image sharpness was calculated as the inverse of the distance between 0.8 (*I*_max_ − *I*_min_) + *I*_min_ and 0.2 (*I*_max_ − *I*_min_) + *I*_min_ [20]. The image sharpness was obtained by averaging over the four profiles.

## 3. Results

**Figure 4:**
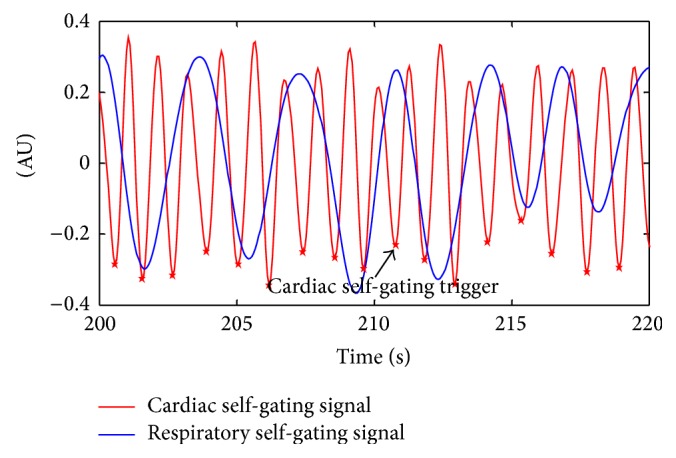
Cardiac self-gating signal (red line) and respiratory self-gating signal (blue line) were synchronized and presented, respectively. Amplitudes of both curves were rescaled for display purposes only. The asterisk represents cardiac self-gating trigger. Note: AU: arbitrary unit.

**Figure 5:**
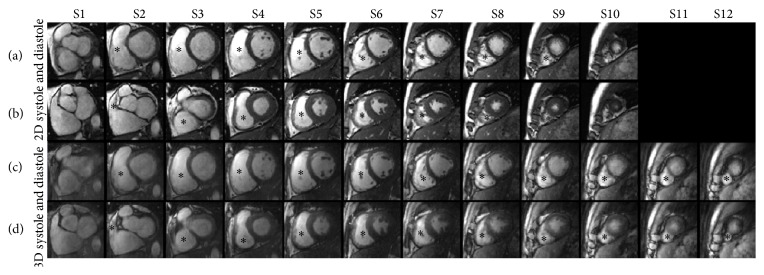
Comparisons of cine images acquired with the standard breath-hold 2D and the proposed free-breathing 3D techniques. Cardiac short-axis images of end-diastolic phase and end-systolic phase obtained with breath-hold 2D technique are shown in (a) and (b), and those obtained with the 3D technique are shown in (c) and (d), respectively. The asterisk denotes the RV.

All scans were completed successfully.  Figure 4 shows an example of synchronized self-gating signals. Temporal resolutions of both self-gating signals were 61.6 ms. The valleys of cardiac self-gating curves were detected and used as trigger. The mean of heart rate and respiratory rate of the subject shown in Figure 4 is 55 bpm (beats per minutes) and 17 bpm (breaths per minute).  Figure 5 shows the short-axis cardiac cine images obtained with breath-hold 2D and free-breathing 3D imaging during diastole and systole, demonstrating similar visualization of cardiac structures and excellent motion suppression of the developed self-gated 3D pulse sequence. Note that 3D imaging yielded 12 contiguous slices without gap, while 2D imaging only provided 10 slices with a 3 mm interslice gap. The 3D images have 61.6 ms temporal resolution and 1.2 × 1.2 mm^2^ in-plane spatial resolution, while the 2D images have 84 ms temporal resolution and 1.2 × 1.6 mm^2^ measured in-plane spatial resolution.

**Figure 6:**
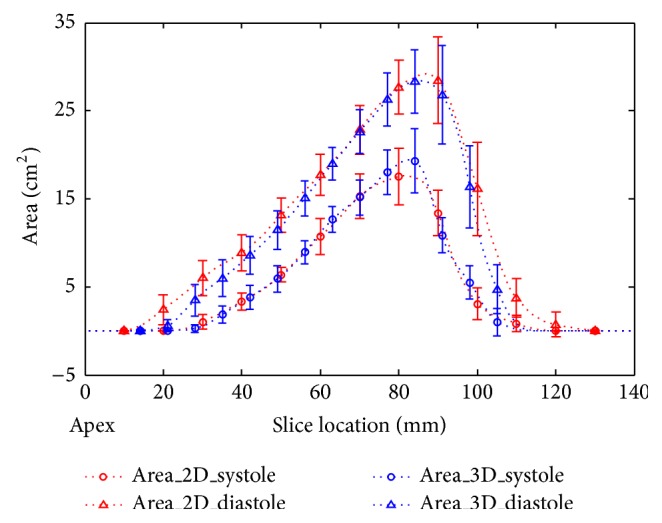
Comparison of systolic and diastolic RV areas obtained at different slice locations using breath-hold 2D and self-gated free-breathing 3D cine imaging.

Figure 6 shows the comparison of RV areas in different slice locations between breath-hold 2D and self-gated free-breathing 3D methods. Average RV diastole and systole areas are shown as red curves for 2D and blue curves for 3D. RV systolic area curves of 2D and 3D fit well and the volumes are similar, with bias of −1.1 ± 6.4 mL (as shown in Table 1). Compared with systole, diastolic areas measured in 2D cine images are larger, especially towards the cardiac apex and base, which causes 2D RVEDV to be higher than 3D with bias of 15.1 ± 8.5 mL, as shown in Table 1.

**Figure 7:**
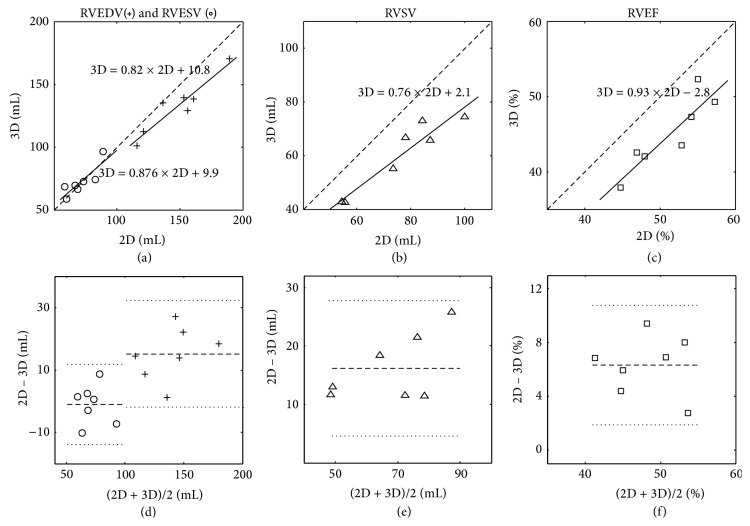
Linear regression and Bland-Altman analysis of RV quantification using 2D and 3D cine MRI. (a)–(c): RVEDV and RVESV, RVSV, and RVEF measurements from seven volunteers with 3D self-gated free-breathing technique (*y*-axis) versus standard 2D breath-hold technique (*x*-axis), identical lines plotted as solid lines. (d)–(f): Bland-Altman plots of RVEDV and RVESV, RVSV, and RVEF measurements. The central dashed lines show the mean bias and the upper and lower dotted lines show the variation limits (±2 SD).

Figure 7 shows the scatter plots and Bland-Altman plots comparing RV functional parameters obtained with breath-hold 2D and self-gated 3D cine imaging. There was a strong correlation between the two techniques with regard to RVEDV (*r* = 0.94), RVESV (*r* = 0.85), RVSV (*r* = 0.95), and RVEF (*r* = 0.89). The Pearson correlations were statistically significant (*P* < 0.05) (Table 1). The linear regression plots (Figures 7(a)–7(c)) show that 3D functional results are linear with 2D. The Bland-Altman plots (Figures 7(d)–7(f)) reveal that differences between 2D and 3D fall within ±2 SD. Table 1 summarizes RV measurements over all subjects. Compared to 2D, there is a reduction of RVEDV by 10%, increase of RVESV by 1.8%, reduction of RVSV by 21%, and reduction of RVEF by 12% in 3D. The difference of the functional measurements between 2D and 3D techniques is statistically significant in RVEDV, RVSV, and RVEF (*P* < 0.05) and not significant in RVESV (*P* = 0.74).

**Figure 8:**
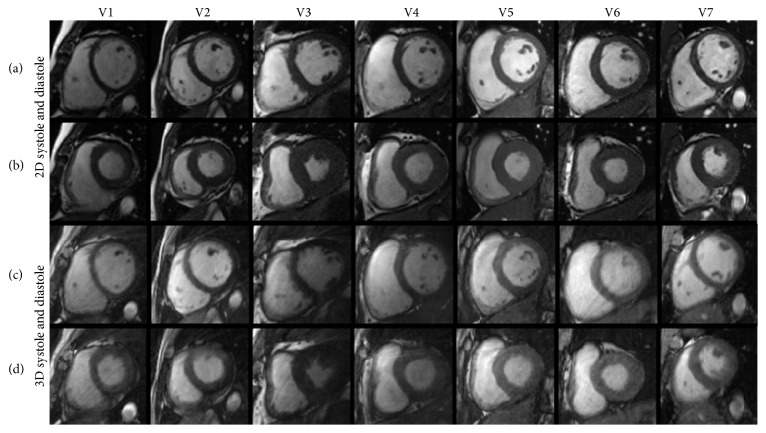
Images of mid-ventricular slices obtained with 2D and 3D cine imaging during end-diastole (a, c) and end-systole (b, d) in seven healthy volunteers.

Figure 8 shows the comparison of mid-ventricular slices obtained during end-diastolic and end-systolic cardiac phases from seven volunteers. A few streaking artifacts appear in 3D self-gated images due to undersampling (undersampling ratio: 2~3) and motion.  Table 2 shows image quality measurements from mid-ventricular images in Figure 8. Compared with 2D, the 3D image quality measurements show a small reduction in blood SNR, myocardium-blood CNR, myocardium contrast, and image sharpness. Compared with other image quality measurements, differences of myocardium-blood CNR and contrast measurements in end-diastole images were statistically significant (*P* < 0.05).

## 4. Discussion and Conclusions

In this study, a respiratory and cardiac self-gated free-breathing 3D cardiac cine imaging method was demonstrated to provide comparable image quality and correlated RV functional parameters to those obtained with the standard breath-hold 2D acquisition in 7 healthy volunteers. These data demonstrate that free-breathing 3D cine MRI can comprehensively assess RV structure and function. The proposed technique that derives respiratory and cardiac self-gating motion signals from original image data is feasible.

RVESV values between the two techniques were similar, with a difference of 1.8% between 3D and 2D, while RVEDV for 3D was 10% lower than it was for 2D. The volumes were calculated from the RV areas of each slice, which were demonstrated in Figure 5. Except for small differences around middle slices, which were mainly caused by bigger areas and slice gaps around the slices, the RV systole areas between 2D and 3D coincide well. On the contrary, the RV diastole area difference was found not only in middle slices but also in apex and basal slices. Besides, the difference of RVEDV was statistically significant (*P* = 0.02). Therefore, the difference between the two methods was not due to segmentation error. Because there are more slices obtained in the diastole phase, gaps between slices in 2D (3 mm) will increase the measurement error, especially around apical and basal slices. Differences between both methods may also be attributable to differences in temporal resolution or misregistration between 2D and 3D cine images of the apical and basal RV. Higher spatial resolution in slice direction could minimize the measurement error. Future study is necessary to improve spatial resolution in slice-direction of 3D cine MRI and test performance for RV assessment in routine clinical practice.

The 2D multiple breath-hold method is usually regarded as the gold standard for cardiac functional measurement. However, it suffers from slice misregistration and therefore has limited accuracy in cardiac chamber volume quantification. The 3D free-breathing self-gated method provides strongly correlated RV functional measurements compared to the 2D technique (Figure 6). However, significant differences were found between the RVEDV, RVSV, and EVEF results. Since it is difficult to choose between the two methods in accurate functional evaluation, other techniques are needed. 3D echocardiography has been shown to be accurate and reproducible for cardiac function measurements [21, 22]. Further study comparing these three techniques would be useful.

The radial trajectory based 3D *k*-space sampling method causes streaking artifacts when data is undersampled, which was found in the RV blood pool in Figures 7(c) and 7(d). In the fixed total scan time of 5 min, 5000 profiles were sampled. Each cardiac phase was assigned a smaller number of profiles (~150) when the respiratory self-gating window was chosen to be 50%. Tornado temporal filter (Figure 3) could remove streaking artifacts using substantial cardiac phases at the cost of increasing cardiac motion blurring. Streaking artifacts may be removed using nonlinear inverse reconstruction [23]. 3D imaging generally provides higher SNR than 2D imaging in radial trajectory. However, thick slab saturation in 3D imaging reduces the inflow effect, so blood SNR and blood-to-myocardium CNR usually degrade when compared to 2D imaging [24]. Therefore, image quality measurements in Table 2 were smaller in the 3D method than in the 2D. In addition, lower SNR and CNR may also be caused by streaking artifacts presented as background noise. The myocardium-blood contrast directly reflects the contrast of two tissues omitting the background noise. However, the myocardium-blood contrast in 3D was still lower than in 2D and statistically significant in end-diastole images. The lower image sharpness in 3D is mainly due to respiratory motion and temporal filtering. Iterative image reconstruction was presented to decrease streaking artifacts and improve image quality [25] compared to regridding reconstruction. Future study is necessary to optimize the image reconstruction method and improve image temporal resolution.

Assessment of the RV in the short axis orientation also has important limitations: the position of the pulmonary and tricuspid valves cannot be clearly identified and therefore it is not usually possible to be certain of the basal boundary of the RV. This process requires manual segmentation of the RV endocardium which previous studies have shown to have a low reproducibility [26, 27]. Yet no further improvements have been reported in recent years and the reproducibility of RV manual RV segmentation remains lower than that of the LV [28]. A reproducibility study of 3D self-gated cine MRI is also needed.

In conclusion, free-breathing 3D cine SSFP imaging was achieved with simultaneous respiratory and cardiac self-gating at SA view for assessment of RV structure and function. Compared with 2D breath-hold method, the 3D RV functional measurements show a reduction of RVEDV by 10%, increase of RVESV by 1.8%, reduction of RVSV by 21%, and reduction of RVEF by 12%. High correlations between the two techniques were found (RVEDV: 0.94; RVESV: 0.85; RVSV: 0.95; and RVEF: 0.89). Compared with 2D, the 3D image quality measurements show a small reduction in blood SNR, myocardium-blood CNR, myocardium contrast, and image sharpness. The 3D SA cine imaging with the proposed technique provides image quality and functional measurements comparable to those acquired with the multiple breath-hold 2D Cartesian SSFP technique.

## Figures and Tables

**Table 1:** RV functional measurements obtained with the standard breath-hold 2D and self-gated free-breathing 3D cine MRI (*N* = 7).

	RVEDV (mL)	RVESV (mL)	RVSV (mL)	RVEF (%)
2D	147.5 ± 25.3	71.4 ± 11.4	76.1 ± 16.7	51.3 ± 4.7
3D	132.4 ± 22.1	72.5 ± 11.7	59.9 ± 13.4	45.0 ± 4.9
Bias	15.1 ± 8.5	−1.0 ± 6.4	16.2 ± 5.8	6.3 ± 2.2
Correlation	0.94	0.85	0.95	0.89
*P* value (Pearson)	0.001	0.02	0.001	0.007
*P* value (Wilcoxon)	0.02	0.74	0.02	0.02

Note: RVEDV: right ventricular end-diastolic volume, RVESV: right ventricular end-systolic volume, RVSV: right ventricular stroke volume, and RVEF: right ventricular ejection fraction.

**Table 2:** Image quality measurements of the standard breath-hold 2D and self-gated free-breathing 3D cine MRI (*N* = 7).

	SNR_Blood_	CNR_Blood-Myocardium_	Contrast_Blood-Myocardium_	Image sharpness (mm^−1^)
2D diastole	105.1 ± 46.1	78.0 ± 29.2	61.9 ± 9.9	0.32 ± 0.03
3D diastole	90.0 ± 20.4	59.7 ± 12.9	49.8 ± 10.5	0.30 ± 0.08
*P* value	0.24	0.03	0.02	0.06
2D systole	104.7 ± 63.5	72.8 ± 44.3	55.4 ± 10.7	0.31 ± 0.04
3D systole	87.8 ± 9.3	60.0 ± 11.5	52.4 ± 12.5	0.23 ± 0.06
*P* value	1.00	0.61	0.61	0.50
